# Medicinal Plants from Northeastern Brazil against Alzheimer's Disease

**DOI:** 10.1155/2017/1753673

**Published:** 2017-02-21

**Authors:** Alexandre Batista Penido, Selene Maia De Morais, Alan Bezerra Ribeiro, Daniela Ribeiro Alves, Ana Livya Moreira Rodrigues, Leonardo Hunaldo dos Santos, Jane Eire Silva Alencar de Menezes

**Affiliations:** ^1^Centro de Ciências Sociais Saúde e Tecnologia, Curso de Enfermagem, Universidade Federal do Maranhão, Rua Urbano Santos, s/n, Centro, 65900-410 Imperatriz, MA, Brazil; ^2^Departamento de Química, Laboratório de Química e Produtos Naturais, Universidade Estadual do Ceará, Campus do Itaperi, Av. Dr. Silas Munguba 1.700, 60.714-903 Fortaleza, CE, Brazil; ^3^Centro de Ciências Sociais Saúde e Tecnologia, Curso de Engenharia de Alimentos, Universidade Federal do Maranhão, Rua Urbano Santos, s/n, Centro, 65900-410 Imperatriz, MA, Brazil; ^4^Núcleo de Pesquisa em Sanidade Animal, Universidade Estadual do Ceará, Campus do Itaperi, Av. Dr. Silas Munguba 1.700, 60.714-903 Fortaleza, CE, Brazil; ^5^Laboratório de Química e Produtos Naturais, Universidade Estadual do Ceará, Campus do Itaperi, Av. Dr. Silas Munguba 1.700, 60.714-903 Fortaleza, CE, Brazil; ^6^Centro de Ciências Sociais Saúde e Tecnologia, Curso de Licenciatura em Ciências Naturais/Biologia, Universidade Federal do Maranhão, Rua Urbano Santos, s/n, Centro, 65900-410 Imperatriz, MA, Brazil; ^7^Centro de Ciências e Tecnologia, Universidade Estadual do Ceará, Campus do Itaperi, Av. Dr. Silas Munguba 1.700, 60.714-903 Fortaleza, CE, Brazil

## Abstract

Alzheimer's disease (AD) has been linked with oxidative stress, acetylcholine deficiency in the brain, and inflammatory processes. In the northeast region of Brazil, various plants are used to treat several diseases associated with these processes; then an antioxidant test was performed with those plants in a previous work and twelve species with higher antioxidant activity were selected for AChE inhibition evaluation. The phenolic compounds content was determined by Folin–Ciocalteu test and flavonoid content with AlCl_3_ reagent using UV-visible spectrophotometry. The antioxidant activity was assessed analyzing the inhibitory activity against 2,2-diphenyl-1-picrylhydrazyl (DPPH) and 2,2-azinobis-3-ethylbenzothiazoline-6-sulfonate (ABTS) and by the *β*-carotene/linoleic acid system and acetylcholinesterase inhibition using qualitative and quantitative tests. The combination of better acetylcholinesterase inhibitory and antioxidant activities pointed out six species, in descending order, as the best potential sources of therapeutic agents against AD:* Hancornia speciosa > Myracrodruon urundeuva > Copaifera langsdorffii > Stryphnodendron coriaceum > Psidium guajava > Mangifera indica*. Besides, the phenolic compounds in the species probably contribute to these activities. However, further pharmacological studies to assess the specific applications of these plants against AD are required to confirm these results.

## 1. Introduction

AD was first described in 1906 by the German physician Alois Alzheimer. It is the most common form of dementia and affects 24 millions of people worldwide. During the progression of AD, the destruction of brain cells leads to gradual memory loss, deterioration of intellectual function, loss of speech, disorientation, and difficulty walking. Factors involved in AD include acetylcholine deficiency, free radicals, and brain tissue inflammation. There is currently no cure for AD, but several drugs aimed at delaying its progression are available, and new and more effective drugs are being investigated [1].

A promise approach in the treatment of Alzheimer's disease (AD) is the use of natural products, which increase acetylcholine levels necessary for memory function [2]. Acetylcholine is a neurotransmitter that is essential for healthy memory, and mental health problems that involve memory loss are directly or indirectly related to acetylcholine. Low levels of this neurotransmitter have been shown to have an important role in the pathogenesis of AD [3]. As a result, many studies aimed at finding treatments for memory changes, such as those that occur in AD, are focused on acetylcholine. AChEIs inhibit acetylcholinesterase, the enzyme responsible for the breakdown of acetylcholine. Studies on plants with acetylcholinesterase inhibitory activity are currently underway, with the aim of discovering new active compounds that are less costly and have fewer adverse side effects than synthetic drugs [4].

Free radicals also a play a role in many common diseases, including AD. Therefore, plant extracts with antioxidant activity also have potential therapeutic value. For example, lipid peroxidation is a reaction mediated by free radicals, in which fatty acid breakdown occurs in cell membranes, thereby reducing membrane fluidity. This process, in association with other events, can lead to pathological conditions such as heart disease, some types of cancer, brain ischemia, and inflammatory conditions [5]. Natural antioxidants obtained from foods, such as phenolic compounds and carotenoids, inhibit lipid peroxidation via their free-radical scavenging activity. The intake of fruits that are rich in phenolic compounds also reduces the risk of developing various types of cancer [6, 7].

A significant reduction in the incidence of chronic and degenerative diseases has been observed in populations with diets comprising mainly natural foods that contain phenolic compounds with antioxidant activity, such as fruits and cereal. The most common antioxidants present in the foods in our diet are vitamin C, vitamin E, selenium, and carotenoids, and in addition phenolic compounds such as phenolic acids and flavonoids also contribute to antioxidant activity [8].

Plants that have positive effects on cognitive disorders, as well as strong acetylcholinesterase inhibitory, anti-inflammatory, and antioxidant activities, are of potential clinical interest for the treatment of AD ([9, 10]).

In this study, an ethnobotanical survey of the main plants used in the northeast region of Brazil was conducted to select medicinal plants that are potential sources of therapeutic agents against AD [11]. Sixty species were found and tested for antioxidant activity. Subsequently, the 34 species that exhibited the highest antioxidant activity were selected, and their phenol and flavonoid content was determined. The plants that yielded the best results were then subjected to acetylcholinesterase inhibition tests. The present study assessed the relationships between these properties in the plants, with the aim of discovering new phytotherapics that can be used in the treatment of AD.

## 2. Materials and Methods 

### 2.1. Collection and Preparation of Plant Extracts

The ethnobotanical and ethnopharmacological study was approved on October 1, 2014 (approval number: 814.666) and was conducted between October 2014 and March 2015. The aim of the study was to investigate some of the main plant species used for medicinal purposes in the northeast region of Brazil, especially with regard to memory. The species were identified morphologically by Professor Ana Zélia Silva, Department of Pharmacy of the Federal University of Maranhão, and were deposited in the Atticus Seabra Herbarium (Table 1). The plant samples were dried, ground in cutting mills, macerated with 70% ethanol for 7 days, filtered, evaporated in a rotary evaporator under reduced pressure, frozen, and freeze-dried. All of the plant names are listed at http://www.theplantlist.org.

### 2.2. Phytochemical Analysis

#### 2.2.1. Determination of Total Phenol Content

Total phenol content was determined using the Folin–Ciocalteu method, with some modifications [12]. The sample was dissolved in methanol, and Folin–Ciocalteu reagent, distilled water, and 15% sodium carbonate were added. After incubation in the dark for 2 hours, the absorbance was measured at 750 nm using a UV/VIS spectrophotometer. The results are expressed as the gallic acid equivalent per gram of extract (mg GAE/g) based on a linear equation for a standard curve prepared with gallic acid (0.1–0.5 mg/mL).

#### 2.2.2. Determination of Flavonoid Content

The flavonoid content was determined using the method described by Funari and Ferro [13]. A volume of 2 mL of the extract solution (2 mg/mL) was used, to which 1 mL of 2.5% AlCl_3_ solution was added. After incubation in the dark for 30 minutes, the absorbance was measured at 425 nm. A yellow color indicated the presence of flavonoids. The flavonoid content was calculated and expressed in mg of quercetin equivalent per gram of extract (mg EQ/g), based on a standard curve prepared with quercetin.

#### 2.2.3. Assessment of Antioxidant Activity via DPPH Radical Reduction

Antioxidant activity was assessed using a previously described method [14], with some modifications. Dilution series of the samples and control, in methanol, were prepared, to obtain the following concentrations: 3.125, 6.25, 12.5, 25.0, 50.0, and 100 *μ*g/mL. Then, 2 mL of DPPH (0.004%) was added. Methanol was used as a negative control. After a period of 30 minutes in the dark, the absorbance was measured at 517 nm using a UV-VIS spectrophotometer. The percentage inhibition was calculated according to the equation: IP% = [(absorbance of DPPH − absorbance of the extract)/absorbance of DPPH] × 100. The IC_50_ values were determined by linear regression of the plotted data.

#### 2.2.4. Assessment of Antioxidant Activity by the ABTS Method 

Antioxidant activity was assessed using the ABTS method, as described by Re et al. [15]. The ABTS solution (7 mM, 5 mL) was mixed with 88 *μ*L of potassium persulfate (140 mM), agitated, and kept in the dark at room temperature for 16 hours. Then, 1 mL of this solution was added to 99 mL of ethanol, and the absorbance was measured at 734 nm. A series of solutions of the plant extracts with decreasing concentrations was prepared, and 3.0 mL of ABTS solution was added to 30 *μ*L of these solutions after 6 minutes. The absorbance was then measured at 734 nm. The IC_50_ values were calculated by linear regression.

#### 2.2.5. Assessment of Antioxidant Activity by the *β*-Carotene/Linoleic Acid Method

The *β*-carotene/linoleic acid method was used according to Wettasinghe and Shahidi [39] in which 1 mg of *β*-carotene was diluted in 5 mL of chloroform. Aerated water, 200 *μ*L of Tween 40, 20 *μ*L of linoleic acid, and 2 mL of *β*-carotene were used to prepare the *β*-carotene solution. The spectrophotometer was adjusted to a wavelength of 470 nm. For the stock solution, 12.5 mg of sample was dissolved in 25 mL of methanol. After the final concentrations were obtained, the *β*-carotene solution (different concentrations) and the blank were placed in cuvettes. The absorbance was measured; then the solutions were placed in a water bath for 2 hours, and the absorbance was measured again. The calculation was performed using the following formula: IC% = 1 − [(absorbance of the sample − absorbance of the sample after 2 hours)/(absorbance of the blank − absorbance of the blank after 2 hours)] × 100. The IC_50_ values were calculated by linear regression.

#### 2.2.6. Determination of Acetylcholinesterase Inhibitory Activity

The acetylcholinesterase inhibitory activity was qualitatively assessed using the method by Ellman et al. [40] adapted for thin layer chromatography [41] and was quantitatively measured using an Elisa BIOTEK microplate reader (model ELX 800 with Gen5 V2.04.11 software), based on the method by Ellman et al. [40] modified by Trevisan et al. [42].

#### 2.2.7. Statistical Analysis

All of the experiments were performed in triplicate, and the results were expressed as the mean ± standard deviation. The differences between the values were examined using analysis of variance (ANOVA), and the results were compared using the Tukey test at a 95% confidence level. GraphPad Prism software version 5.01 was used.

## 3. Results and Discussion 

The present study began with an ethnobotanical investigation of 60 species, to identify active agents that may be used in the treatment of several diseases, including AD [11]. AD is one of the most prevalent neurodegenerative diseases in humans, and its cause is multifactorial, but free radicals and acetylcholinesterase are known to be strongly associated with this disease. Therefore, the aim of this study was to analyze the main medicinal plants used in the northeast region of Brazil and determine their antioxidant activities, phenolic compound content, and flavonoid content, and acetylcholinesterase inhibitory activity.

Phenolic compounds, flavonoids, and tannins are the major antioxidant agents and free-radical scavengers in plants. Several methods can be used to assess the antioxidant activity of a sample; however, one method alone cannot provide precise enough data to determine a plant's free-radical scavenging or prevention capacity or the consequent amount of lipid oxidation, especially in plant extracts that are composed of several chemical compounds that act via different mechanisms [43]. As a result, in this study, the species were tested using the DPPH, ABTS, and *β*-carotene/linoleic acid methods.

Table 1 identifies the main species assessed in this study. Their scientific and common names are given, along with their voucher numbers, the parts of the plant typically used, and their traditional uses and biological and pharmacological reports.

Table 2 shows the results of antioxidant tests, phenols, and flavonoid content an AChE inhibition. In bold, six plant species were marked since they were chosen as being better candidates as anti-AChE agents. This choice took into account the combination of the two kinds of actions:* H*.* speciosa*,* C. langsdorffii*,* M. urundeuva*,* M. indica*,* P. guajava*, and* S coriaceum*.

The species that exhibited the best antioxidant activity in the three tests and anticholinesterase activity was* H. speciosa.* This plant is currently used in the treatment of several conditions, including fractures, inflammation, ulcers, pain, hypertension, and diabetes, and its effects are probably attributable to the antioxidant capacity of compounds present, such as phenols, tannins, flavones, flavonoids, leucoanthocyanidins, and alkaloids [44].* H. speciosa* also exhibited the best results among all the species tested in terms of anticholinesterase activity. This activity, combined with its high content of phenols and flavonoids, means* H. speciosa* may be the best source among the assessed species for research into new therapeutic agents against several diseases, including AD.

*B. forficata* is currently used as a hypoglycemic in the treatment of diabetes. Recent studies have demonstrated that insulin metabolism is important in the AD signaling pathway, and there is evidence that alteration of insulin metabolism plays a role in the molecular pathogenesis of AD [45]. Patients with AD have lower levels of insulin, and when these levels are corrected, there is an improvement in cognitive processes [4], further supporting the idea that* B. forficata *has potential in the treatment of AD. This plant also had good anticholinesterase activity and a high antioxidant capacity and therefore could be another source of agents against AD.* B. forficata* also exhibits a high flavonoid content, which confirms that this species is a potential source of promising natural resources for studies on new AD treatment strategies [46].

*C. langsdorffii *and compounds isolated from it are reported as gastric mucosal protective agents that act by increasing the production of mucus [47]. Because this species exhibited high acetylcholinesterase inhibitory activity, this mechanism may contribute to the plant's ability to increase acetylcholine, a neurotransmitter involved in several biological processes, including the production of mucus, healing, inflammation, and gastric ulcers.

*M. indica* exhibits the highest flavonoid content among the species tested, as well as high antioxidant activity, which probably contributes to its anti-inflammatory properties [30], and high anticholinesterase activity, as was demonstrated in the present study. These data confirm the results of other studies with this species, which have shown it to have anticholinesterase, anti-inflammatory, antioxidant, and antidiabetic activities. These pharmacological activities probably result from the phenolic compounds that have been isolated in this species, such as mangiferin, and suggest that* M. indica *is another promising source of agents for the treatment and prevention of AD [48]. Recent studies on cholinergic dysfunction, oxidative stress, and their relationship with memory have demonstrated that ethanol extracts of the fruits of* M. indica *improved memory function [49].* M. indica* fruit extracts have potential neuroprotective activity and improve cognitive impairment, because they reduce oxidative stress and increase cholinergic function [49].

*M. urundeuva* bark tinctures are largely used as anti-inflammatory phytotherapics and the action is explained due to the levels of tannins and dimeric chalcones [50]. However, there are few studies on the effects of this species on the central nervous system. Extracts of* M. urundeuva* were used in animal models of Parkinson's disease and were found to reverse behavioral changes and increase the number of neurons and their viability. At the neurochemical level, these extracts prevented the reduction of dopamine level, because dopamine levels are low in Parkinson's disease, and probably promote neuroprotection via their antioxidant and anti-inflammatory activities, and these results demonstrate that this plant is useful in the treatment of neurodegenerative diseases [51].* M. urundeuva *were shown to have excellent acetylcholinesterase inhibitory activity, a high phenol content, and high antioxidant activity which shows that* M. urundeuva *also has potential as a source of therapeutic agents against AD.

*P. guajava* also exhibited high antioxidant activity and had a high content of phenolic compounds and flavonoids, in accordance with a previous study, and it was found to be useful in alleviating various oxidative stress-related diseases [52]. The antidiarrheal activity of the plant is probably due to the high phenolic content.

*S. coriaceum *exhibits acetylcholinesterase inhibitory activity, as well as antioxidant, anti-inflammatory, antimicrobial, healing, antiulcerogenic, and leishmanicidal activities [35, 36]. The* S. coriaceum* extract was shown to have excellent acetylcholinesterase inhibitory and antioxidant activities, which make it another potential source of therapeutic agents against AD.

## 4. Conclusions

The assessed natural products are promising sources of pharmacological agents for the treatment of AD, which affects 24 million people worldwide. The strategy chosen seems to be a good choice by selecting plants, which are inhibitors of acetylcholinesterase, and also possesses antioxidant activity. Phenols and flavonoids are important natural products that inhibit acetylcholinesterase and thus restore acetylcholine level essential for brain function; therefore the six medicinal species selected are promising sources of natural products that can be used in studies for discovering new therapeutic compounds against AD.

## Figures and Tables

**Table 1:** Selected medicinal plants from Northeastern Brazil with high antioxidant activity.

Species [family] Local name	Voucher number	Part used	Traditional use	Biological and pharmacological activities
*Anadenanthera peregrina *(L.) Speg. [Fabaceae] Angico	1423	Bark	Expectorant, flu, bronchitis, asthma, cough	Anti-inflammatory [16]

*Bauhinia forficata* Link [Fabaceae] Mororó	1322	Bark	Diabetes, kidney problems	Hypoglycemic, antioxidant, anti-inflammatory, diuretic [17–19]

*Copaifera langsdorffii* Desf. [Fabaceae] Copaiba	1400	Bark	Inflammation, wound healing	Anti-inflammatory, antioxidant, healing, antimalarial, leishmanicidal [20–23].

*Euterpe oleracea* Mart [Arecaceae] Açaí	1425	Seed	Memory, high blood pressure, general illness	Antioxidant, anti-inflammatory, antinociceptive, antihypertensive [24–26]

*Hancornia speciosa* Gomes [Apocynaceae] Mangaba	1399	Bark	Wound healing, inflammation, stomach ulcers, stomach pain	Antihypertensive, antioxidant, anti-inflammatory [27, 28]

*Luehea divaricata* Mart. & Zucc [Malvaceae] Açoita cavalo	1117	Leaf	Inflammation, diabetes, irregular menstruation	Anti-inflammatory, antinociceptive, immunostimulatory [29]

*Mangifera indica* L. [Anacardiaceae] Manga	1213	Leaf	Headache, ulcers, fever	Antiulcerogenic, antioxidant, anti-inflammatory [30]

*Myracrodruon urundeuva* Allemão [Anacardiaceae] Aroeira-Sertão	1420	Bark	Inflammation, vaginal infection, sore throat, wound healing	Anti-inflammatory, healing [31]

*Plathymenia reticulata* Benth [Fabaceae] Candeia	1414	Bark	Inflammation, liver problems, bleeding	Anti-inflammatory, antihemorrhagic, antibiotic, antioxidant, antivenom [26, 32]

*Psidium guajava* L. [Myrtaceae] Goiaba	1182	Leaf	Diarrhea	Antidiarrheal, antioxidant [33, 34]

*Stryphnodendron coriaceum* Beth [Fabaceae] Barbatimão	1033	Leaf	Vaginal infection, toothache, wound healing, inflammation	Anti-inflammatory, antimicrobial, healing, antiulcerogenic, antileishmanial, antioxidant [35, 36]

*Syzygium aromaticum* (L.) Merr. & L. M. Perry [Myrtaceae] Cravo da India	1428	Fruit	Menstrual colic, pain, sedative, inflammation	Antioxidant, antidiabetic, analgesic, anti-inflammatory [37, 38]

**Table 2:** Antioxidant activity (DPPH, ABTS, and *β*-carotene/linoleic acid tests), phenolics content, flavonoid content, and acetylcholinesterase inhibitory activity of selected medicinal plant species used in the northeast region of Brazil.

Species	DPPH *μ*g/mL	*β*-Carotene *μ*g/mL	ABTS *μ*g/mL	Phenols Mg GAE/g extract	Flavonoids mg EQ/g extract	AChEI IC_50_ *μ*g/mL
Ap	53.65 ± 0.24^f^	12.51 ± 0.66^ed^	2.12 ± 0.01^a^	62.23 ± 0.15^b^	0.88 ± 0.04^j^	397.23 ± 0.09^i^
Bf	49.39 ± 0.47^e^	13.72 ± 0.22^ef^	3.44 ± 0.01^c^	38.03 ± 0.53^h^	4.62 ± 0.03^c^	10.90 ± 1.44^a^
Cl	45.23 ± 0.17^d^	13.74 ± 0.99^ef^	2.64 ± 0.02^b^	48.03 ± 0.30^g^	4.35 ± 0.01^d^	14.86 ± 1.79^b^
Eo	28.54 ± 0.78^a^	22.09 ± 0.81^h^	2.04 ± 0.17^a^	51.10 ± 0.59^f^	1.31 ± 0.06^g^	130.29 ± 0.37^g^
Hs	30.70 ± 0.68^b^	5.73 ± 0.38^b^	2.18 ± 0.06^a^	60.26 ± 0.45^c^	8.64 ± 0.02^b^	10.13 ± 0.17^a^
Ld	38.48 ± 0.53^c^	10.77 ± 0.55^dc^	3.98 ± 0.11^d^	55.81 ± 0.39^d^	0.83 ± 0.04^i^	111.54 ± 0.33^f^
Mi	50.78 ± 0.47^e^	1.18 ± 0.16^a^	2.13 ± 0.05^a^	28.79 ± 1.16^i^	13.51 ± 0.04^a^	29.67 ± 0.42^d^
Mu	48.01 ± 0.35^e^	6.92 ± 1.42^b^	2.14 ± 0.02^a^	61.28 ± 0.44^bc^	0.96 ± 0.03^hi^	10.75 ± 0.15^a^
Pr	51.33 ± 0.34^e^	19.67 ± 0.36^g^	3.93 ± 0.21^d^	61.56 ± 0.27^bc^	0.91 ± 0.01^i^	46.93 ± 0.76^e^
Pg	40.42 ± 0.81^c^	15.06 ± 0.40^f^	2.22 ± 0.06^a^	37.85 ± 0.53^h^	3.82 ± 0.02^e^	18.98 ± 0.11^c^
Sc	45.09 ± 0.15^d^	13.71 ± 0.21^ef^	1.96 ± 0.05^a^	52.85 ± 0.02^e^	1.05 ± 0.01^h^	17.56 ± 0.50^c^
Sa	56.54 ± 0.99^g^	8.81 ± 0.26^c^	4.00 ± 0.07^d^	91.27 ± 0.26^a^	2.02 ± 0.01^f^	152.25 ± 0.04^h^
Ru	39.20 ± 0.16^c^	9.25 ± 0.01^c^	2.05 ± 0.08^a^	—	—	—
Es	—	—	—	—	—	19.53 ± 0.08^c^

The data are presented as the mean ± standard deviation and were analyzed using ANOVA followed by a Tukey test. Values with different superscript letters are significantly different (*P* < 0.05). Flavonoid content: mg EQ/g extract; phenolic compounds content: mg GAE/g extract; 2,2-diphenyl-1-picrylhydrazyl (DPPH), 2,2-azinobis-3-ethylbenzothiazoline-6-sulfonate (ABTS), *β*-carotene/linoleic acid, and acetylcholinesterase inhibition (AChEI): IC_50_ (*μ*g/mL). Ap, *Anadenanthera peregrina* (bark); Bf, *Bauhinia forficata* (bark); Cl, *Copaifera langsdorffii* (bark); Eo, *Euterpe oleracea* (seeds), Hs, *Hancornia speciosa* (bark); Ld, *Luehea divaricata *(leaves); Mi, *Mangifera indica* (leaves); Mu, *Myracrodruon urundeuva* (bark); Pr, *Plathymenia reticulata* (leaves); Pg, *Psidium guajava* (leaves); Sc, *Stryphnodendron coriaceum* (bark); Sa, *Syzygium aromaticum *(fruits); Ru, rutin (antioxidant control); Es, eserine (acetylcholinesterase control); —: not performed.
